# p-BioSPRE—an information and communication technology framework for transnational biomaterial sharing and access

**DOI:** 10.3332/ecancer.2014.401

**Published:** 2014-02-11

**Authors:** Gabriele Weiler, Christina Schröder, Fatima Schera, Matthias Dobkowicz, Stephan Kiefer, Karsten R Heidtke, Stefanie Hänold, Iheanyi Nwankwo, Nikolaus Forgó, Martin Stanulla, Cornelia Eckert, Norbert Graf

**Affiliations:** 1 Fraunhofer Institute for Biomedical Engineering IBMT, Ensheimer Strasse 48, 66386 St. Ingbert, Germany; 2 Fraunhofer Institute for Biomedical Engineering IBMT, Branch Potsdam-Golm, Am Mühlenberg 13, 14476 Potsdam-Golm, Germany; 3 Leibniz Universität Hannover, Institut für Rechtsinformatik, Königsworther Platz 1, 30167 Hannover, Germany; 4 Universitätsklinikum des Saarlandes, Kirrberger Straße, 66421 Homburg, Germany; 5 Pediatric Haematology and Oncology, Hannover Medical School, Carl-Neuberg-Straße 1, 30625 Hannover, Germany; 6 Department of Paediatric Oncology/Haematology, Charité Universitätsmedizin Berlin, Campus Virchow Klinikum, Augustenburger Pl. 1, 13353 Berlin, Germany.

**Keywords:** biobank access, specimen management, p-BioSPRE, ObTiMA, Trial Biomaterial Manager, IDB, p-biobank wrapper

## Abstract

Biobanks represent key resources for clinico-genomic research and are needed to pave the way to personalised medicine. To achieve this goal, it is crucial that scientists can securely access and share high-quality biomaterial and related data. Therefore, there is a growing interest in integrating biobanks into larger biomedical information and communication technology (ICT) infrastructures. The European project p-medicine is currently building an innovative ICT infrastructure to meet this need. This platform provides tools and services for conducting research and clinical trials in personalised medicine. In this paper, we describe one of its main components, the biobank access framework p-BioSPRE (p-medicine Biospecimen Search and Project Request Engine). This generic framework enables and simplifies access to existing biobanks, but also to offer own biomaterial collections to research communities, and to manage biobank specimens and related clinical data over the ObTiMA Trial Biomaterial Manager. p-BioSPRE takes into consideration all relevant ethical and legal standards, e.g., safeguarding donors’ personal rights and enabling biobanks to keep control over the donated material and related data. The framework thus enables secure sharing of biomaterial within open and closed research communities, while flexibly integrating related clinical and omics data. Although the development of the framework is mainly driven by user scenarios from the cancer domain, in this case, acute lymphoblastic leukaemia and Wilms tumour, it can be extended to further disease entities.

## 1. Introduction

A research-integrated biobanking solution is crucial for the advent of personalised medicine for cancer patients. Technological progress in cancer-related molecular biology and molecular imaging has led to an exponential increase in the possibilities for individualised cancer care. Moreover, the accompanying complexity of the resulting biomarkers and their application in, for instance, radiotherapy planning and targeted drug choice and dosing has made the knowledge base too complex for physicians and certainly incomprehensible for patients. Many more new potential biomarkers will be discovered in the next years, and their validation as useful biomarkers in diagnosis and therapy will require fast reference to the global collection of well-characterised and annotated human biospecimens. Beyond access to high-quality biological samples, research will also require integrated access to sample-related clinical and omics data of increasing granularity.

To address this need, a biobank access framework called ‘p-medicine Biospecimen Search and Project Request Engine’ (p-BioSPRE) has been designed and integrated into a general information and communication technology (ICT) infrastructure for personalised medicine, the p-medicine platform, aiming to cut therapy development time from bench to bedside [1]. The p-medicine platform is currently developed in the European project p-medicine [2] to foster research and clinical decision support for personalised therapy. This platform provides tools and services to semantically integrate and analyse heterogeneous biomedical data in a data warehouse. It also comprises an ontology-based trial management system called ObTiMA [3] to design and conduct clinical trials. Within the p-medicine platform, the p-BioSPRE biobank access framework and the p-medicine data warehouse are foreseen to complement each other and synergise to generate and contribute critical Virtual Physiological Human knowledge.

The aim of p-BioSPRE is to provide access to different kinds of human biomaterials and related data for research purposes. A biobank operator is supported in offering his biomaterial and related data to research communities, while safeguarding donors’ personal rights and enabling biobanks to keep control over the donated material and related data. A researcher is enabled to search the biomaterial that is offered within his communities. It is furthermore possible for him to request biomaterial for a specific research project and his request is forwarded online to the biobank operator.

For managing biomaterial and interlinking the p-medicine data warehouse and p-BioSPRE, the ObTiMA Trial Biomaterial Manager is developed. It enables users of ObTiMA, the p-medicine’s ontology-based trial management system, to manage their biomaterial data within clinical trials. For this purpose, a pre-defined but adjustable case report form (CRF) for patient’s biomaterial is provided in ObTiMA. The biomaterial data can be integrated with clinical data and exported to p-BioSPRE as detailed below.

## 2. Methods

To build an integrated biobanking solution for p-medicine, two typical biobanking user scenarios have been analysed and user requirements identified. Furthermore, different tools and projects for integrated biobanking have been evaluated with the aim to find models, guidelines, or software that can be adapted to the biobanking user scenarios and constitute the base of an integrated biobanking framework. Based on these analyses, a biobank access framework has been developed in close interaction with end users and legal experts.

### 2.1. User scenarios

The technical development of the biobank access framework is mainly driven by two user scenarios provided by p-medicine partners in the context of Wilms tumour trials and clinical research, and trials related to acute lymphoblastic leukaemia (ALL). These scenarios constitute typical use cases for biobanking in a clinical trial setting, for any disease area whatsoever. Therefore, solutions based on these use cases will be generic and applicable in a wider biomedical research context.

The International Society of Paediatric Oncology (SIOP) [4] is responsible for conducting successive international multicentric Wilms tumour trials across Europe. Within these trials, biomaterial is collected in different participating hospitals and sent to a central national biorepository, where the material is stored and further analysed. Related biomaterial data are also stored at the central biorepository.

Currently biomaterial and data stored in the central biorepositories are not accessible via an information system from the participating hospitals or the study centre (central facility where the trial is coordinated). Clinical and biobanking data cannot be integrated since the data are stored in different incompatible systems. The data are only accessible on-site and sharing of biomaterial between international research partners is a very time-consuming procedure, e.g., in the German SIOP trial, the central biorepository is located at the Biocenter of the University Würzburg. Data about the biomaterial are captured in several spreadsheets in Würzburg, e.g., analysis information or information about the quality of the biomaterial. Clinical data are collected in the web-based trial management system ObTiMA that is hosted at the study centre at the University Homburg, e.g., information about the patient’s clinical data including chemotherapy, surgery, and radiotherapy, but also pathology data and outcome data. Biomaterial data stored in the spreadsheets and clinical data stored in ObTiMA cannot be integrated automatically into one platform. In addition, there is no query tool available that allows searching for specific biomaterial samples. Questions like ‘How many samples are available from female patients that have stage I tumours having relapsed and having not relapsed?’ are not resolvable so far. Even access to the data and further statistical analysis of the data cannot be done by the study centre in Homburg, as long as these data are stored in independent databases.

The International BFM Study Group (I-BFM SG) [5] is dedicated to promote both research and clinical care for patients with leukaemia and lymphoma. A growing number of participating national study groups from meanwhile more than 30 countries worldwide collaborates in multicentric trials in the field of leukaemia and lymphoma, e.g., around 80% of childhood ALL patients in Germany are included in ALL-BFM trials, which are coordinated by the University Hospital Schleswig-Holstein of the Christian-Albrecht-Universität zu Kiel. If ALL patients, who were treated in an ALL-BFM study, relapse, they are usually treated according to ALL-REZ BFM protocols. ALL-REZ trials are coordinated by the Charité Universitätsmedizin Berlin.

Currently, BFM partners can only access clinical data and biomaterial data of their own patients stored in their own local databases. In particular, it is not possible for the ALL-REZ BFM study centre in Berlin to access their patients’ biomaterial of initial diagnosis and data in the study centre in Kiel online. Regarding European and worldwide collaborations for research on ALL, it is highly desired to access and share biomaterial and related analytical data within the respective international research community.

Therefore, the following generic needs have been identified for the p-BioSPRE biobank access framework (and beyond):

- The international study groups need to integrate biomaterial repositories to be able to share biomaterial and data within national and international research communities. This is currently not possible but required to foster research in the field of personalised medicine.
  In particular:
  - Biobank operators need to offer their biomaterial and data that are stored in legacy biobank management systems to research communities.
  - Researchers need to search for and request specific human biomaterial that is appropriate for their research.
- It is necessary to integrate biomaterial data with corresponding clinical and omics data preferably over the same information system.

Patients’ privacy and autonomy, as well as researchers’ and biobanks’ confidentiality and autonomy must be safeguarded at any time.

In addition, requirements specific for the SIOP and I-BFM SG communities, including data structure and existing software and database architecture must also be fulfilled in a flexible manner, allowing p-BioSPRE to integrate further (and so far unknown) biobank partners consecutively.

### 2.2. Evaluation of tools and guidelines

There are numerous tools and projects that focus on the integration of biobanks. We have evaluated many different approaches with the aim to find models, guidelines, or software that could be adapted to the biobanking user scenarios described in the last section, and constitute the base of an integrated biobanking framework in the p-medicine platform (see Table 1 and [6] for an overview). To confine adaptation efforts, flexibility and interoperability of these tools with local biobank information management systems, which already pre-exist with use case partners, are of paramount importance. This flexibility was given highest priority for the evaluation summarised in Table 1, and it was found to be fulfilled best by the CRIP tools. Furthermore, we have drawn two main conclusions that we outline in the following.

- Though Biobanking and Biomolecular Resources Research Infrastructure (BBMRI) is not operable yet, it is of outmost importance that the results of the preparatory phase of the BBMRI project [7] be considered when developing p-BioSPRE. BBMRI aims to build a European biobanking research infrastructure, and they have diligently elaborated requirements and criteria for integrated biobanking, which have already been implemented in Nordic countries [8, 9]. It is foreseen that BBMRI will be implemented in the future under the European Research Infrastructure Consortium legal entity. As of 14 October 2013, final approval of this entity by the European Commission is expected for autumn 2013.
- As mirrored by the BBMRI list of requirements (cf. [6] chapter 5.2.2) and confirmed by our analysis of legal interoperability below, a trans-institutional or even transnational biobanking platform can practically only be operated on anonymised data (processing of personal data is only allowed under certain circumstances, see page 20), which should be displayed in statistical groups only. Among all the tools and guidelines, which we have evaluated (Table 1, [6]), only the CRIP concept [10] fulfils this critical requirement. All the other tools and suites, such as the caBIG Tissue Banks [11] or i2b2 [12], are more or less centralised biobank and data management systems processing pseudonymised data. Referring to the European Union (EU) Data Protection Directive (see Section 2.3 below), pseudonymised data are as a general rule to be regarded as personal data (for more information see Art. 29 Working Party, Opinion 4/2007 on the concept of personal data, http://ec.europa.eu/justice/policies/privacy/docs/wpdocs/2007/wp136_en.pdf, pp 18–20), and hence strict requirements of the specific applicable national data protection laws would have to be met, which in effect would amount to a serious obstacle for transnational data transfer.

CRIP is a model for a meta biobank [10] that has already been adapted for a central national German biobanking infrastructure [13, 14], and shortlisted by the STRATUM project as a system for the development of a UK biobank catalogue for biomedical research [15]. Hence, we have selected the CRIP toolbox [16] as a base for p-BioSPRE since it is in line with BBMRI’s recommendations on federated biobanking infrastructures, adaptable to all needs of the user scenarios and furthermore in a productive state since 2006.

### 2.3. Legal interoperability

Apart from the technical aspect of transnational and interoperable biobanking infrastructure, the legal framework is also an important factor to consider when developing a solution to the problems identified above. This is because biobanks and clinical research centres operate under strict rules in terms of biomaterial handling as well as processing of sensitive personal data associated with the biomaterials. These rules are to a large extent not harmonised within the EU, which means that in practice, different legal regimes apply for the use of the samples for research on the one hand, and the processing of associated personal data of the donors on the other hand. As a result of this, any platform that tends to integrate biobanking infrastructures across Europe will have to comply with multiple laws, or at least, aim at fulfilling the requirement of the regime with the highest threshold. Although national laws implementing the Data Protection Directive (Directive 95/46/EC of the European Parliament and of the Council of 24 October 1995 on the protection of individuals with regard to the processing of personal data and on the free movement of such data) are in a more coherent framework (it should be noted that implementing the Data Protection Directive into 28 EU member states laws has also resulted into some remarkable differences in the national data protection laws), there is, however, no such coherent framework on a European level for the sharing of biomaterial.

However, to implement the objectives of the p-BioSPRE meta biobank, relevant national legal regimes implementing the Data Protection Directive, and the biobanking laws were considered. In this regard, one elementary aspect of consideration is the scope of application of national data protection laws. Those only apply to the processing of personal data (see Art. 3 Data Protection Directive), which is defined as: ‘any information relating to an identified or identifiable natural person (‘data subject’); an identifiable person is one who can be identified, directly or indirectly, in particular by reference to an identification number or to one or more factors specific to his physical, physiological, mental, economic, cultural, or social identity’ [Art. 2 lit. (a) Data Protection Directive]. This definition is broad, encompassing every conceivable data that refer to a person. This means that as long as a person can be identified with reasonable means from data that are, for example, held in an electronic patient record, this data are regarded as personal data. All kinds of data, such as clinical data (e.g., clinical laboratory test results, patient demographics, pharmacy information, and radiology reports), data from biomaterial that could be linked to an individual, and so on, will fall into this category. To determine whether a person is identifiable, account has to be taken of all the means likely reasonably to be used either by the controller or by any other person to identify the said person (Recital 26 Data Protection Directive; according to the Art. 29 Working Party, one should in particular consider all the factors at stake: e.g., the cost of conducting identification, the intended purpose, or risks of organisational dysfunctions and technical failures; Opinion 4/2007 on the concept of personal data, viewed 25.11.2013, <http://ec.europa.eu/justice/policies/privacy/docs/wpdocs/2007/wp136_en.pdf>). This broad criterion means that only a narrow segment of data could fall outside the scope of personal data.

Anonymisation of personal data (it is controversial if anonymisation of personal data is to be seen as a form of processing of personal data, see [17] pp. 42–43.) appears to be the only solution that permits processing in complex situations, which consequently eliminates the requirement to comply with the strict standards applicable to processing of personal data (it is even legally required to keep data no longer in a form that permits identification of data subjects for longer than necessary for the purposes for which the data were collected or for which they are further processed, see Art. 6 (e) Data Protection Directive). However, it is not an easy task to achieve such complete anonymity, especially with respect to the fast moving developments in data linking and mining technology. This means that the anonymous status can swap quickly into a personal one. This can only be forestalled by regular evaluations of the risk of reidentification of the data. This risk can be decreased and controlled by a number of technical and organisational measures, such as restricting access and access controls, strong passwords registration procedures, and contractual agreements. To handle these issues, a legal and contractual framework has been developed along with the ICT infrastructure for p-BioSPRE that provides the base for secure and legally compliant biomaterial and related data sharing.

After careful evaluation, we decided that the legal framework setup for the meta biobank will operate with anonymous data. In this case, the strict standards applicable to processing of personal data have not to be considered because the national laws implementing the Data Protection Directive do not apply. In addition, the contractual framework aims at securing this anonymity to achieve a compliant biomaterial and data sharing framework favourable to all jurisdictions. A detailed discussion on the legal and contractual framework is given in Section 3.2.

To enforce patients’ decision on the research use of their specimens, and to prepare for existing privacy regulations [18], p-BioSPRE will be equipped to record tiered informed consent and provide this information, if available, for the meta biobank query.

## 3. Results: the p-BioSPRE biobank access framework

Based upon analysis of the two user scenarios, we have designed the p-BioSPRE biobank access framework to provide registered researchers access to different kinds of human biomaterials and related data. Starting with a minimum dataset (Table 2), the framework provides means to harmonise data (see Section 3.1.2) and facilitates data import by help of a metadata repository (MDR).

Only the six items constituting the minimum dataset (bold text in Table 2) must be mandatorily provided by the biobank partners, keeping the threshold low for them to participate. Although overlap with the MIABIS minimum dataset [19] proposed for BBMRI is small so far (cf. Table 2), it can easily be completed upon the biobank partners’ request. Numerous additional data fields shown in Table 2 emerged from the ALL-BFM and ALL-REZ BFM databases and can at any time be changed or extended without programming efforts over the MDR (cf. Section 3.1.2). Once integrated, all data fields are displayed over the p-BioSPRE search tool (see Section 3.1.1).

In particular, the p-medicine biobank framework supports the following main functionalities:

(a) Offering human biomaterial for research
  A biobank operator is supported in providing data on his biomaterial and related clinical data to open or closed research communities, as defined in p-BioSPRE. Data provision is regulated in p-BioSPRE’s legal framework especially by p-medicine’s Biobank Data Transfer Agreement (see Section 3.2). Any biobank management system can be used to import data into and anonymise data within p-BioSPRE. It is up to the operator to decide upon research communities to whom his data will be disclosed (or not), and upon his response to incoming project requests. This ensures full control over his material and data.
(b) Requesting specific human biomaterial for research purposes
  A registered researcher is enabled to search for and request biomaterial that is available within his research communities. He can access information about data linked to biomaterial and the number of available cases (a case (‘psn’ in Table 2) is defined as the data belonging to a specific disease of a donor/patient) backed by specimens. The p-BioSPRE web portal enables researchers to define a search profile according to the classification and annotations stored in the underlying database. The result of queries is presented as statistical groups, based on a k-anonymised view on the data. The project portal allows selection of statistical groups from the search result. Researchers are then requested to describe the scope of the envisioned project and can specify additional services (e.g., polymerase chain reaction, immunohistochemistry, fluorescence *in situ* hybridisation; see Figure 4, ‘Requested Methods’). Upon submission of the project request, the providing hospital or consortium will be displayed.
(c) Managing biomaterial data in ObTiMA
  Users of ObTiMA, the p-medicine’s ontology-based trial management system, can manage their biomaterial data within clinical trials. For this purpose, a pre-defined but adjustable CRF for patient’s biomaterial is provided in ObTiMA, the so-called biobanking specimen CRF. The biomaterial data can be integrated with clinical data within a trial or across several trials for further analysis. The collected biomaterial data can be shared by uploading it into p-BioSPRE. Legacy biomaterial data can be imported into ObTiMA from excel files.

Altogether, p-BioSPRE is compliant with the aforementioned BBMRI list of requirements for data integration systems ([6] chapter 5.2.2), including (among others)

- compliance with local database policies, national ELSI regulations and EU data protection regulations (R 1);
- user authentication and authorisation (R 9);
- full control of local biobanks on the data they expose (R 14);
- anonymisation/k-anonymisation of data and metadata (R 15, R 16);
- metadata driven query and analysis tool, not custom-written against a fixed data model (R 27).

### 3.1 p-BioSPRE software components

**Figure 1.**
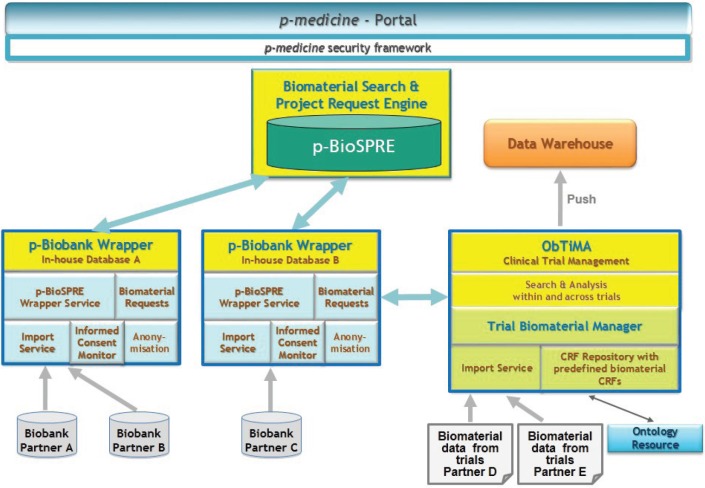
Basic architecture of the p-medicine biobank access framework.

We have designed the biobank access framework as a set of coupled components, which are shown in Figure 1.

The main component of the framework is p-BioSPRE, which is a meta biobank to share biomaterial for research purposes. p-BioSPRE is based on the CRIP meta biobank [10]. Furthermore, the framework comprises the ***p-biobank wrappers***, which are tools to support biobank operators to make their biomaterial and related data available in p-BioSPRE and to manage associated requests, independent from their underlying biobank management system. This is achieved by the core of each p-biobank wrapper, ***the local In-house Database (IDB)***, a component of the CRIP Toolbox [16]. To enable users of the p-medicine trial management system ObTiMA, to integrate biomaterial data in clinical trials, and to offer them in p-BioSPRE (via the p-biobank wrappers), a ***Trial Biomaterial Manager*** is provided.

In the following, we will describe the functionality of the different components in more detail.

#### 3.1.1. p-BioSPRE: the p-medicine biomaterial search and project request engine

p-BioSPRE is a meta biobank that provides researchers the possibility to search for and request biomaterial that fits their research purposes.

Technically, p-BioSPRE is based on the CRIP meta biobank [16]. It is a web application and database architecture and it can be accessed via the p-medicine portal, the main access point of the p-medicine platform.

**Database architecture**

From the biobank operators' local IDBs (see Section 3.1.2) data are uploaded to the p-BioSPRE central database. The anonymised data are encrypted via SSL protocol and then uploaded to the central database, which is hosted at Fraunhofer IBMT. Before upload, biobank operators need to authenticate. In the central database, each subsequent upload will completely replace the previous one of this biobank partner ensuring that cases (cf. Table 2) cannot be traced back. Although search in the central database is performed on anonymised cases, the result is in addition displayed as pools of cases/statistical data only (see text below and Figure 3). This design delivers, through a differentiated query of granular, yet anonymised data, a comprehensive and satisfying result (Figures 3 and 4) to the p-BioSPRE user, though compliant with all privacy and data protection requirements (cf. Section 3.2).

**Figure 2.**
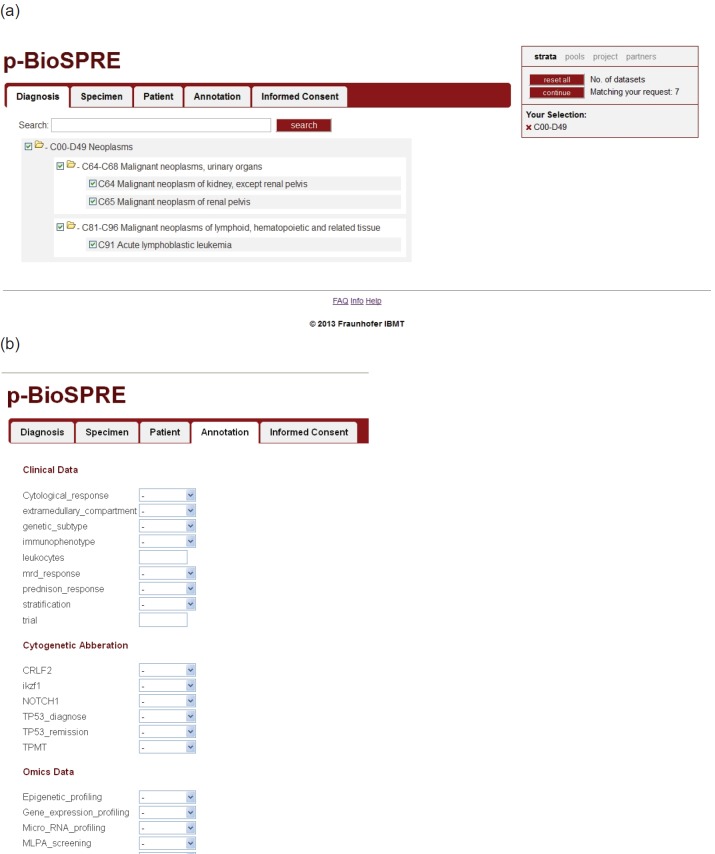
p-BioSPRE search tool. (a) Search criteria can be entered under five tabs representing multiple options each. Selected criteria are shown in the box ‘Your selection’, overall number of available matching cases just above. (b) Annotation of specimens includes clinical, cytogenetic, and omics data. The selection of parameters shown here is representing the ALL dataset and can be extended for any other datasets if required.

**p-BioSPRE search tool**

p-BioSPRE provides a search interface that enables authorised users to search for biomaterial. After authentication, users access an interactive search tool (Figure 2) allowing to select:

- **diagnosis** (based on ICD 10 and ICD-O);
- **specimen** (type of specimen, e.g., whole blood, serum, tissue (FFPE or cryo-preserved), DNA, RNA, etc.);
- **patient data** (age, sex, body mass index (BMI), etc.);
- **annotation** (including clinical information and genetic subtypes);
- **consent** (information on patient’s informed consent given for the underlying biobank/trial).

Although initially only cases of Wilms tumour and ALL will be processed in p-medicine, the system is open to integrate and display all diagnoses, as technically provided by the CRIP concept.

**Figure 3.**
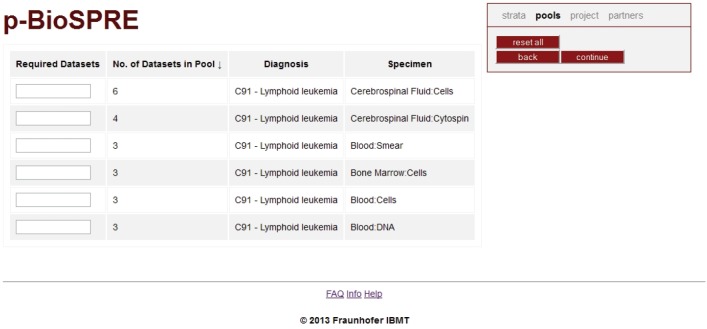
p-BioSPRE pool list. Upon ‘continue’, the pools selected by requesting a number of cases (‘Required Datasets’, left column) are automatically inserted into the Project Request Form (Figure 4). Note that pools shown in this screenshot are small since to date only 50 datasets have been imported into p-BioSPRE. Display of minimum number of datasets in a pool can be set by default (see Section 3.1.2, elements of k-anonymisation).

Once the user has selected appropriate criteria (‘strata’, cf. Figure 2a) for his envisioned project, the ‘continue’ button will open up the list of ‘pools’ (Figure 3) or, in other words, of (statistical) groups of cases matching the user’s request. Although providing the user with the sought-after information how many cases and specimens would be available for his/her project, tracking of single cases or patients will obviously not be possible over the p-BioSPRE search tool. Hence, the statistical (or ‘aggregated’) data shown on the p-BioSPRE pool list are fully in line with data and privacy protection requirements. This design allows a maximum protection of privacy-related interests of donors, in particular anonymity of donors within the p-BioSPRE infrastructure, whereas at the same time access to information on available specimen is provided.

(a) Search criteria can be entered under five tabs representing multiple options each. Selected criteria are shown in the box ‘Your selection’, overall number of available matching cases just above. (b) Annotation of specimens includes clinical, cytogenetic, and omics data. The selection of parameters shown here is representing the ALL dataset and can be extended for any other datasets if required.

**Figure 4.**
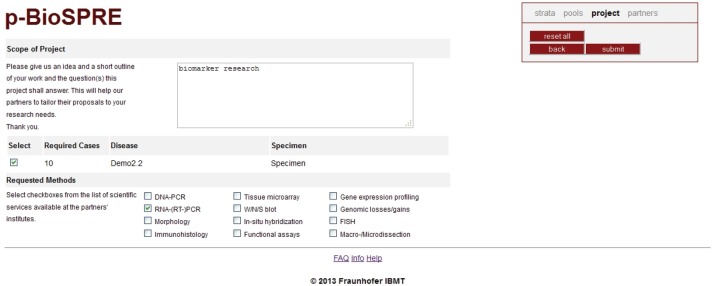
p-BioSPRE project request form. Submission of the ‘Project Request Form’ generates a file with the search profile that is automatically conveyed to the participating biobank partners. Requested biobanks will run this ‘input file’ on their local IDB (Figure 5; button ‘Project request’) and retrieve a list of pseudonymised cases matching the project request.

Upon ‘continue’, the pools selected by requesting a number of cases (‘Required Datasets’, left column) are automatically inserted into the Project Request Form (Figure 4). Note that pools shown in this screenshot are small since to date only 50 datasets have been imported into p-BioSPRE. Display of minimum number of datasets in a pool can be set by default (see Section 3.1.2, elements of k-anonymisation).

**Figure 5.**
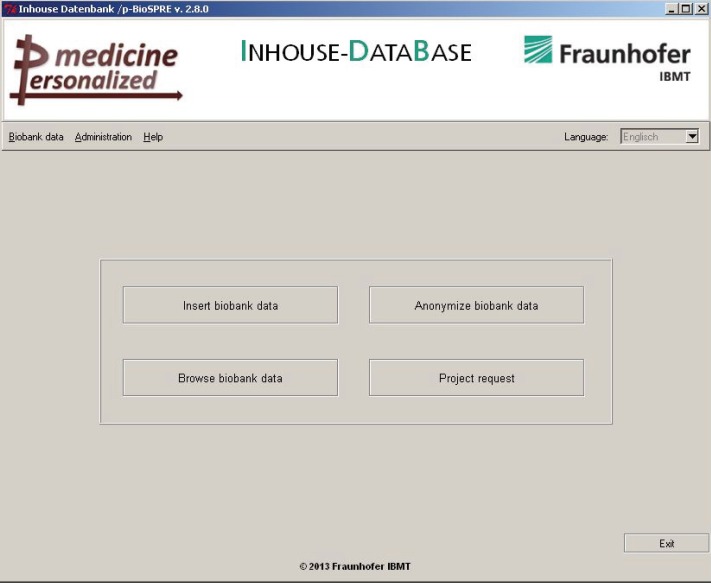
p-Biobank wrapper user interface: management of IDB. Functionalities of buttons are as follows: 1) insert biobank data: select and import a data file; 2) anonymise biobank data: deidentify data and create a file ready for export to p-BioSPRE; 3) browse biobank data: View cases (e.g., to restrict export); and 4) project request: execute a project request (file) and retrieve cases matching the request.

Submission of the ‘Project Request Form’ generates a file with the search profile that is automatically conveyed to the participating biobank partners. Requested biobanks will run this ‘input file’ on their local IDB (Figure 5; button ‘Project request’) and retrieve a list of pseudonymised cases matching the project request.

When a user has found appropriate biomaterial for his research, he can request it over an interactive request form (Figure 4) enabling him to specify the amount of biomaterial he needs and to outline his research project in some detail (i.e. as far as relevant for the biobank). Having submitted his project request, a receipt window will open up to the user indicating the biobank operator(s) to whom his request has been forwarded. He is then free to contact them directly or await their response within ten workdays, as agreed over the Biobank Data Transfer Agreement (see Section 3.2, ‘The request procedure’).

Functionalities of buttons are as follows: 1) insert biobank data: select and import a data file; 2) anonymise biobank data: deidentify data and create a file ready for export to p-BioSPRE; 3) browse biobank data: View cases (e.g., to restrict export); and 4) project request: execute a project request (file) and retrieve cases matching the request.

p-BioSPRE forwards the request as an xml file to the biobank operator enabling him to quickly retrieve the requested material over his/her p-biobank wrapper. In other words, the result of the project request tool is a *search profile* that can be applied on the data stored in the IDBs of the p-biobank wrapper, delivering the list of locally available cases matching the user’s project request/search criteria. The biobank can then contact the researcher and decide about the request.

#### 3.1.2. p-Biobank wrappers

Biomaterial data are uploaded into p-BioSPRE from so-called p-biobank wrappers. Technically, a p-biobank wrapper is based on the IDB, a component of the CRIP toolbox that comprises a local database, software, and web services that can be installed at the biobank operator’s site.

A p-biobank wrapper provides biobank operators a comfortable user interface to share their biomaterial and related data in p-BioSPRE (cf. Figure 5).

It imports pseudonymised data on biomaterial and provides a selected, anonymised export file to p-BioSPRE for online queries (Figure 5, buttons ‘Insert biobank data’ and ‘Anonymise biobank data’). Upon import, data are harmonised (e.g., by unifying different formats for time stamps or for sample donor’s sex (male/female; m/f, etc.), and deidentified, e.g., by stripping off identifiers or pseudonyms and by converting a patient’s exact age into full years. Further features of the the p-biobank wrappers are

- elements of k-anonymisation (see below) [20];
- calculation of values (e.g., age of patient from dates of birth and sample preservation, or of BMI from patient’s height and weight);
- MDR.

Because of the MDR, p-biobank wrappers can easily be adapted to the export functionality of the biobank information management system: the MDR just needs to be configured accordingly without any programming efforts.

The user model of the p-biobank wrappers provides roles for

- one or more biobank(s)/user(s) (import/export/search data);
- an administrator (delete data from database/add users/add biobanks).

Data will not be exported automatically to p-BioSPRE, export can only be triggered by the participating biobank operators (cf. Figure 5, button ‘Anonymise biobank data’). Thus, before data will be sent to the p-BioSPRE database, all identifying data will be stripped off, and no personal information will be transferred.

**Elements of k-anonymisation**

Some p-BioSPRE features towards an approach for k-anonymity, or to depersonalise data at least by widening the interval that is being processed or displayed for this data, have already been mentioned in the text above: A patient’s exact age is always converted into full years already upon import into the p-biobank wrapper. In addition, over the p-BioSPRE search tool, a minimum of five-year intervals may be selected for sample donor’s age.

As shown in the legend of Figure 3, a minimum number of datasets in a pool can be set by default in the search tool, and in the IDB as well. In the search tool, this would prevent any pools below a certain limit from being displayed. In the IDB, this feature allows implementing specific requirements of local Ethics Review Boards prescribing that a pool must contain, for example, a minimum of five cases to be processed, safeguarding that projects based on a smaller number of cases cannot be performed.

#### 3.1.3. ObTiMA trial biomaterial manager

The Trial Biomaterial Manager is developed as a component of the web based trial management system of the p-medicine infrastructure ObTiMA [3]. It enables management of biobanks and associated specimen data in clinical trials and sharing selected specimen data.

The Trial Biomaterial Manager provides users an interface to manage specimen data in clinical trials. For this purpose, a predefined biobanking specimen CRF that allows users to collect different kind of specimen information is provided that can be adjusted to the user’s needs. This CRF is stored in the ObTiMA CRF Repository [3], a repository for reusable patient CRFs.

An interface is provided to get an overview of the available biomaterial. Furthermore, it is possible to link clinical data and biomaterial data within clinical trials.

Optionally, a biobank can share selected data on biomaterial by uploading it via the p-biobank wrapper anonymised to p-BioSPRE. In the following, the current functionality of the Trial Biomaterial Manger is described in more detail.

**Managing biobanks**

The Trial Biomaterial Manager provides biobank operators the possibility to create virtual biobanks, edit and view the biobank metadata, and manage the samples of the biobanks. Furthermore, biobank operators can assign trials to the biobank and specify and control, which ObTiMA users may access the data of the biobank.

**Figure 6.**
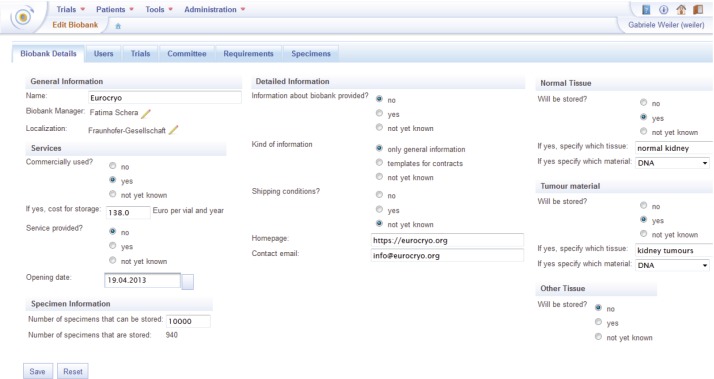
ObTiMA Trial Biomaterial Manager.

In Figure 6, the user interface to edit the metadata of a biobank is shown. The metadata can be edited under several tabs. In the tab ‘Biobank Details’, the main metadata for the biobank can be filled in by the user. The biobank details comprise:

- General Information as the name, the biobank operator, and the location of the biobank.
- Specification of the provided services, e.g., the specification of the storage cost and if the biobank is used commercially.
- Specimen information as the number of specimens that can be stored and the number of specimens that are being stored at present.
- Detailed information: additional information about the biobank, e.g., the homepage and the contact mail.
- Tissue information: information about the tissue types stored in the biobank.

Furthermore, the user can list the members of the biobank committee. It is possible to assign biobanks to trials in order to enable collection of biomaterial for the biobank in different trials. In ObTiMA, specimens are created in trials to simplify the integration with the clinical data collected in the trial. A specimen that is created in a trial can be assigned to a specific biobank, but only if the biobank has been previously assigned to the trial. The assignment of the biobank to the trial needs to be confirmed by the trial chairman.

A biobank operator can also assign ObTiMA users permissions to manage the biobank, e.g., editing metadata for the biobank or viewing specimens. A user with the latter permission can only view specimens that are stored in the biobank. By clicking on a specimen, the user is directed to the CRF in the appropriate trial, where the specimen has been created and can access clinical as well as specimen data of the patients.

**Creating specimens and biobanking specimen CRF**

**Figure 7.**
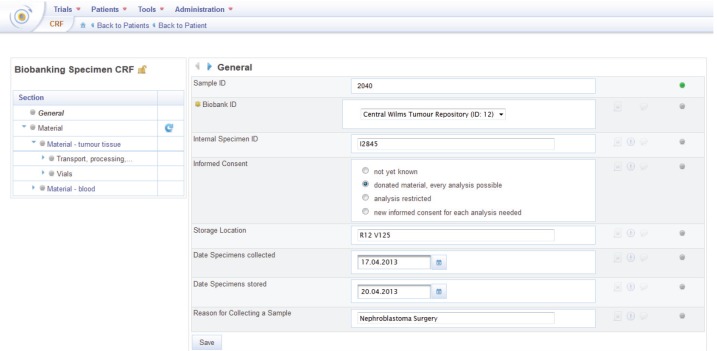
Biobanking specimen CRF.

A specimen can be created by assigning the biobanking specimen CRF to a patient (Figure 7). This CRF is stored in the ObTiMA CRF Repository. On this CRF, relevant information describing a biobank sample can be collected on items that are structured into the following sections and subsections:

- General: general information about the specimen, e.g., information about informed consent or storage location.
- Material: material type, e.g., blood or tumour tissue, and depending on the material type items to characterise the material, e.g., the total volume of collected blood or the available volume for material type blood.
  - Transport, processing, and storage: quality information about the transport, processing, and storage, e.g., the processing method or the freezing temperature.
  - Vials: information about the available vials, e.g., when the material in the vials was isolated.
    - Analyses: information about the analyses made with the specimen, e.g., information about the normalisation and the used platform.

A precondition to create a specimen in a trial is adding the biobanking specimen CRF to the trial in the design phase and adapt it to the trial’s needs. For this purpose, ObTiMA provides a user-friendly interface that allows adding, changing, and/or deleting items on a CRF.

The mechanism to store sample data on CRFs simplifies the integration with clinical data that are as well stored on patient CRFs. It is possible to explore patient’s biobanking and clinical data in ObTiMA. Furthermore, the data can be exported in one file for further analysis.

**Shipping biomaterial**

The Trial Biomaterial Manager supports also the process of shipping biomaterial samples from a study centre to a biobank operator. The biobank operator must not know the identifying data of the patient. Therefore, when shipping the biomaterial, the study centre needs to send the pseudonym to the biobank operator to enable matching the referenced patient in ObTiMA. Barcode labels are used to facilitate handling of the very long pseudonyms. For this purpose, a study centre can print a barcode for the patient and label the specimen for transport to the biobank operator. In turn, the biobank operator can find the referenced patient by simply scanning the barcode.

I**nterface to p-BioSPRE and the p-medicine data warehouse**

Interfaces to p-BioSPRE and the p-medicine data warehouse are provided (cf. Figure 1). A trial chairman or biobank operator can export the specimen data of a trial to make it available in p-BioSPRE. To this end, the pseudonymised biobanking data can be exported into a file in CDISC ODM format [21], a standard format for exchanging trial data and seamlessly upload it into a p-biobank wrapper installation. The Trial Biomaterial Manager integrates push services that are used to push selected biomaterial data into the p-medicine data warehouse. Here, the data can be seamlessly integrated with heterogeneous biomedical data, e.g., clinical or microarray data, and subsequently it can be analysed with the tools provided by the p-medicine platform. Thus, the push services interlink the p-medicine data warehouse and p-BioSPRE.

### 3.2. Legal framework for biomaterial sharing

Alongside the ICT framework, a legal framework has been provided for p-BioSPRE to ensure compliance with applicable regulations. This framework deals with data protection and data security issues within the p-BioSPRE meta biobank, and regulates the relationship between the actors: meta biobank operator, biobank operators, and researchers. In the following, we describe the most important aspects of this legal framework.

**Data protection and data security**

National data protection laws implementing the Data Protection Directive have been considered when developing the p-BioSPRE meta biobank architecture. This is important because processing of personal data is prohibited unless informed consent of the concerned person has been obtained or a national provision, implementing the Data Protection Directive, allows such data processing (Art. 7 and 8 (Art. 8 Data Protection Directive include health data or data revealing racial or ethnic origin as sensitive data) of the Data Protection Directive). It is usually difficult to fulfil the rigorous requirements associated with processing of sensitive personal data, and where permissible, researchers resort to using anonymous data, that is, data considered as not identifying any person, although it is still controversial whether certain types of data, such as genetic data, can be truly anonymous [22]. However, anonymous data would not be considered as ‘personal data’ in the sense of Art. 2 lit. (a) of the Data Protection Directive, with the consequence that the Directive and the implementing national laws on processing of personal data do not apply (See Section 2.3).

On the basis of the above, the data flow within the meta biobank infrastructure has been designed so that p-BioSPRE processes only anonymised data, and displays statistical data that do not reveal any individual identity when performing a search. Both measures are complemented by introducing elements of k-anonymity as described above (see. Section 3.1.2).

In addition, a Biobank Data Transfer Agreement between the meta biobank operator and the biobank operator has also been introduced. This agreement includes an obligation on the meta biobank operator to import only anonymised data, and to refrain from reidentifying or matching datasets to reveal individual identities. In addition, a p-medicine meta biobank access policy has been developed as part of the organisational measure to control the context in which data are processed. In essence, access to the meta biobank is restricted to only registered researchers whose identity, institutional affiliations, and research interests have been verified by the p-medicine meta biobank operator. To register, researchers need to download a form, fill it in, and send it to the meta biobank operator. With the latter, staff (four-eye principle) will check the registration form for completeness and plausibility and document their approval before assigning password and username to the applicant.

Furthermore, confidentiality and security are guaranteed by an advanced security solution that relies on the p-medicine security framework. The p-medicine security framework is based on open commonly used stable standards, such as SAML 2.0 [23], SSL, and X.509 (X.509 is a standard for public key infrastructure: http://datatracker.ietf.org/wg/pkix/charter/). The framework provides features, e.g., brokered authentication for services and an extended roles and rights system. It restricts access only to authenticated users who have sufficient access rights and ensures that all communication is encrypted through SSL.

A detailed description of the p-medicine security framework can be found in [24]. The integration of the p-medicine security framework into the legal framework for p-BioSPRE is described in [25]. In this document, it is especially outlined in detail how p-BioSPRE uses the brokered authentication of p-medicine via a gateway solution to guarantee secure access only for authorised users.

**Informed consent**

The nature, design, and scope of consent that was obtained during the collection of biosamples are part of the critical factors in allowing access to the samples stored in biobanks. It is of utmost importance that the consent form has clear rules and conditions of sharing data and biosamples to avoid a future problem in reusing the samples for other researches. Researchers need to be aware of the secondary use-problem limiting research and data processing for a purpose that was not foreseen when collecting the sample and data. This problem, however, goes beyond the legal framework developed for p-BioSPRE. The framework here covers access to data about existing biomaterials only. The exchange of physical material is not part of the overall framework because in practice, it is subjected to bilateral material-transfer agreements between the biobanks and the requesting researchers.

Data on informed consent (“IC data”) are integrated into p-BioSPRE (cf. Figure 2). This will on the one hand enable biobanks to internally, check whether the consent will cover a requested research project, enabling them to inform external researchers as early as possible if the request will be granted or not.

**The request procedure**

Researchers will also have the opportunity to request for material over the meta biobank portal. This request will be forwarded to the responsible biobank operator, thus facilitating the negotiation process for material transfer. The biobank operator and the researcher then need to agree on the conditions under which the samples will be provided considering the applicable national legislations. The p-medicine Biobank Data Transfer Agreement includes clauses on the procedure and time frame for response to the request by biobank operators. For example, it is envisaged that an answer to the access request shall be given in reasonable time, not later than ten working days after receiving the request though the p-medicine meta biobank. A detailed description of the legal framework for p-BioSPRE can be found in [25].

## 4. Conclusion

p-medicine’s biobank access framework p-BioSPRE is an integrated biobank solution to support research in personalised medicine. It enables and facilitates secure transnational access to existing biobanks, but also offering biomaterial collections to research communities. Biobank specimens can be managed using the ObTiMA Trial Biomaterial Manager. Along with biobank data, information on patients’ informed consent is also processed and displayed. The development of p-BioSPRE is driven by two user scenarios on ALL and Wilms tumour, to seamlessly adapt the framework to the needs of integrated biobanking.

The biobank access framework is integrated into the p-medicine platform, enabling that biobanking data can be integrated with heterogeneous biomedical data, e.g., clinical or microarray data, and subsequently can be analysed with the tools provided by the p-medicine platform.

An initial version of the biobank access framework is available on a test server of the p-medicine portal, the main access point of the p-medicine platform, and can be evaluated by interested research communities under the URL https://pmedportal.ibmt.fraunhofer.de. In the next months, it will be evaluated by the SIOP and the ALL study groups. Components of the framework will then be further adapted to user needs and improved with additional features.

## Conflicts of interest

The authors have declared that no competing interests exist.

## Authors’ contributions

All the authors wrote the manuscript and agreed on the final version.

## Figures and Tables

**Table 1.** ICT tools for integrated biobanking (overview; see [6] for further details).

Tool/Acronym	URL	Characteristics	Aspects regarding p-BioSPRE
caTissue Suite	https://cabig.nci.nih.gov/tools/catissuesuite, accessed 31/10/2011 and 27/11/2013	NCI initiative; centralised sample inventory/manual annotation; open source	Questions on user friendliness and maturity raised by evaluation report^a^. Anonymisation questionable
BBMRI Preparatory Phase; WP 5	http://www.bbmri.eu/index.php/workpackages/wp-5, accessed 31/10/2011	EU—FP 7 project aiming to build a pan-European biobanking research infrastructure	Concepts, but no tools available
i2b2	http://www.i2b2.org/, accessed 31/10/2011 and 27/11/2013	‘Informatics for Integrating Biology and the Bedside’; open source	Adaptation for biobank access required. Changes and exchanges have to deliver to the Brigham and Women’s Hospital Inc.
SIMBioMS	http://simbioms.org/; accessed 31/10/2011 and 27/11/2013	Web-based open-source software system for managing data and information in biomedical studies; SAIL subsystem for biobank access.	Deeper technical information needed. SAIL provided under the GNU GPL licence: Implications for p-BioSPRE exploitation
P3G DataSHaPER	http://www.datashaper.org/; accessed 31/10/2011 and 27/11/2013	Tools to foster interoperability of epidemiological cohorts and population-based biobanks	Epidemiological tool: adaptation to disease-oriented biobanks would be required
CRIP Toolbox	http://www.crip.fraunhofer.de/; accessed 31/10/2011 and 27/11/2013	Proprietary tools of Fraunhofer IBMT; operative since 2006 and approved by German data protection authorities	Tools available, operative, and tested since 2006. Modularity of software allowing for flexible solutions in different settings. Anonymisation/privacy regime approved by German data protection authorities

BBMRI: Biobanking and Biomolecular Resources Research Infrastructure.

p-BioSPRE: p-medicine Biospecimen Search and Project Request Engine.

EU: European Uninon.

a http://deainfo.nci.nih.gov/advisory/bsa/bsa0311/caBIGfinalReport.pdf; accessed 27/11/2013.

**Table 2.** Minimum dataset (in bold) and metadata (in alphabetical order) of p-BioSPRE^a^

Description	Name	Type
Age of donor^b^	age	int
Biobank acronym	biobank	text
Name of contact person (7)	biobank_contact	text
e-mail address of contact person (9)	biobank_email	text
Name of biobank (2)	biobank_name	text
	blasts_bm	text
	blasts_pb	text
	crlf2	text
	cytological_response	text
Date of birth (full years only)	dateofbirth	date
Date of sample preservation	dateofsamplepreparation	date
Diagnosis	diagnosis	text
**Class of code**	**diagnosis_class**	**text**
**Code of diagnosis**	**diagnosis_code**	**text**
	epigenetic_platform	text
	epigenetic_profiling	text
	exom_sequencing_platform	text
	extramedullary_compartment	text
	gene_expression_platform	text
	gene_expression_profiling	text
	genetic_subtype	text
	genome_sequencing_platform	text
**Group of material**	**groupofspecimen**	**text**
	ikzf1	text
	immunophenotype	text
	labeldateofbiopsy	text
	leukocytes	text
	localisation	text
	localisation_class	text
	localisation_code	text
	micro_rna_platform	text
	micro_rna_profiling	text
	mlpa_kit	text
	mlpa_screening	text
	mrd_response	text
	notch1	text
**Patient**	**ppsn**	**text**
	prednison_response	text
**Case**	**psn**	**text**
Sex of donor	sex	text
	snp_diagnose	text
	snp_platform_diagnose	text
	snp_platform_remission	text
	snp_remission	text
	stage	text
	stratification	text
	tp53_diagnose	text
	tp53_remission	text
	tpmt	text
	trial	text
	trial_follow_up	text
**Material type (41);**	**typeofspecimen**	**text**
	whole_exome_seq	text
	whole_genome_seq	text

a aMinimum data include: diagnosis_class (standard used, e.g., ICD-O or ICD-10); diagnosis_code (actual code, e.g., C64 for malignant neoplasms of kidney); group of specimen (e.g., blood, tissue); ppsn (patient); and psn (case). A patient can constitute several cases, and a case can be backed by several specimen types and aliquots. Where applicable, attribute numbers as listed by MIABIS [19] are given in brackets behind the description of data fields.

b calculated from year of birth and year of sample preservation.
